# Cognitive Reserve Assessment Scale in Health (CRASH): Its Validity and Reliability

**DOI:** 10.3390/jcm8050586

**Published:** 2019-04-28

**Authors:** Silvia Amoretti, Bibiana Cabrera, Carla Torrent, Caterina del Mar Bonnín, Gisela Mezquida, Marina Garriga, Esther Jiménez, Anabel Martínez-Arán, Brisa Solé, Maria Reinares, Cristina Varo, Rafael Penadés, Iria Grande, Estela Salagre, Eduard Parellada, Miquel Bioque, Clemente Garcia-Rizo, Ana Meseguer, Gerard Anmella, Adriane R Rosa, Fernando Contreras, Gemma Safont, Eduard Vieta, Miquel Bernardo

**Affiliations:** 1 Barcelona Clinic Schizophrenia Unit, Hospital Clinic of Barcelona, Neuroscience Institute, 08036 Barcelona, Spain; amoretti@clinic.cat (S.A.); bcabrera@clinic.cat (B.C.); mezquida@clinic.cat (G.M.); RPENADES@clinic.cat (R.P.); EPARELLA@clinic.cat (E.P.); MBIOQUE@clinic.cat (M.B.); CGARCIA3@clinic.cat (C.G.-R.); AMESEGUA@clinic.cat (A.M.); ANMELLA@clinic.cat (G.A.); 2 Biomedical Research Networking Center for Mental Health (CIBERSAM), 28029 Madrid, Spain; ctorrent@clinic.cat (C.T.); CBONNIN@clinic.cat (C.d.M.B.); magarriga@clinic.cat (M.G.); EJIMENE1@clinic.cat (E.J.); AMARTIAR@clinic.cat (A.M.-A.); BSOLE@clinic.cat (B.S.); REINARES@clinic.cat (M.R.); VARO@clinic.cat (C.V.); IGRANDE@clinic.cat (I.G.); ESALAGRE@clinic.cat (E.S.); fcontreras@bellvitgehospital.cat (F.C.); gsafont@mutuaterrassa.es (G.S.); evieta@clinic.cat (E.V.); 3 University of Barcelona, 08036 Barcelona, Spain; 4 August Pi I Sunyer Biomedical Research Institute (IDIBAPS), 08036 Barcelona, Spain; 5 Bipolar and Depressive Disorders Unit, Institute of Neurosciences, 08036 Barcelona, Spain; 6 Laboratory of Molecular Psychiatry, Hospital de Clínicas de Porto Alegre, Porto Alegre 90035-003, Brazil; adrianerrosa@gmail.com; 7 Postgraduate Program: Psychiatry and Behavioral Science, Universidade Federal do Rio Grande do Sul (UFRGS), Porto Alegre 90040-060, Brazil; 8 Department of Pharmacology and Postgraduate Program: Pharmacology and Therapeutics, Universidade Federal do Rio Grande do Sul (UFRGS), Porto Alegre 90040-060, Brazil; 9 Psychiatry Department, Bellvitge University Hospital-IDIBELL, 08907 L’Hospitalet de Llobregat, Spain; 10 Hospital Universitari Mutua Terrassa, 08221 Terrassa, Spain

**Keywords:** cognitive reserve, cognition, assessment, reliability, validity, severe mental illness, affective disorders, non-affective psychosis, psychosocial functioning

## Abstract

(1) Background: The cognitive reserve (CR) concept has not been precisely defined in severe mental disorders and has been estimated using heterogeneous methods. This study aims to investigate and develop the psychometric properties of the Cognitive Reserve Assessment Scale in Health (CRASH), an instrument designed to measure CR in people with severe mental illness; (2) Methods: 100 patients with severe mental illness (non-affective psychoses and affective disorders) and 66 healthy controls were included. The internal consistency and convergent validity of CRASH were assessed. Spearman’s correlations coefficients were also performed to examine the relationship between CRASH and neuropsychological tests, psychosocial functioning, and clinical course; (3) Results: The internal consistency was high (Cronbach’s alpha coefficient = 0.903). The CRASH global score had a large positive correlation with the Cognitive reserve questionnaire total score (*r* = 0.838, *p* < 0.001), demonstrating good convergent validity. The correlation coefficients between the CRASH total scores and clinical, functional, and neuropsychological performance were different between groups. In order to provide clinical interpretation, severity classification based on diagnosis (non-affective psychotic disorders, affective disorders, and healthy controls) have been created; (4) Conclusions: CRASH is the first CR measure developed specifically for patients with severe mental illness, facilitating reliable and valid measurement of this construct. The scale may aid in the stratification of patients and the implementation of personalized interventions.

## 1. Introduction

The concept of cognitive reserve (CR) has been defined as the ability of the brain to make flexible and efficient use of cognitive networks in order to minimize the clinical manifestation of the pathology of dementia [1]. At the beginning, the concept of CR was developed in the context of aging and dementia. In this field, it is hypothesized that CR includes the capacity to withstand damage, the ability to compensate for damage through the use of alternate networks, and remodelling and plasticity [2]. Stern proposes at least two types of reserve: the first relates to the use of brain structures or networks not normally used in order to compensate for brain damage and the second concerns the use of brain networks or cognitive paradigms that are less susceptible to disruption when coping with task demands (used by healthy individuals as a normal process) [2]. Therefore, this second type could explain the variation in the performance of the healthy individual, particularly when they must perform to the maximum of their capacity [2].

In the field of neuropsychiatric disorders, there have been attempts to associate CR with clinical expression and there is an increasing evidence showing that CR may be considered as a resilience factor [3]. Some studies in patients with a first episode of psychosis (FEP), schizophrenia, or bipolar disorder suggest that high CR is associated with a later onset of psychosis and better recovery, and it is considered to be a positive moderator of the impact of pathology on clinical course, functional outcome, and cognitive performance [3,4,5,6,7,8,9,10,11,12]. However, in mental disorders the way to conceptualize CR remains open to debate. To date, the most common proposed proxies of CR include estimated premorbid intellectual quotient (IQ), educational level, occupational attainment, and leisure activities [4,8,9,10,12]. Nevertheless, CR has been estimated using heterogeneous methods [4,8,9,10], which makes it difficult to compare studies. Therefore, there is a need to create a specific scale for the assessment of this relevant construct. For example, in order to assess the proxy “Education-Occupation”, de la Serna et al. [4] took into account the number of years of obligatory education that subjects had completed; school performance before the beginning of the disorder; parents’ educational level; and questions about the children’s development in terms of language, reading, writing, and motor functions. Instead, Forcada et al. [8] included quantifications of educational and work attainment during youth and adulthood. However, Anaya et al. [9] used the number of years of formal education and the Scale for Occupational Prestige PRESCA-2. In a FEP sample, we included the number of years of completed obligatory education, parent’s educational level, and the life time school performance [10]. Overall, the lack of a standardizing procedure to quantify CR in mental disorders makes it difficult to optimally compare studies.

Different instruments are available to assess CR in both healthy populations and those with dementia, including the “Lifetime of Experiences Questionnaire (LEQ)” [13], “cognitive reserve questionnaire (CRQ)” [14], “CR Index questionnaire (CRIq)” [15] and “cognitive reserve scale (CRS)” [16]. Nevertheless, taking into account that the emergence of a FEP usually occurs during late adolescence or early adulthood (between 15 and 30 years of age), the aforementioned instruments were not specifically created to assess populations with a severe mental illness (SMI) such as a first episode of psychosis (FEP), schizophrenia, or bipolar disorder.

More specifically, the LEQ [13] consists of 42 questions, and it was designed for individuals over the age of 65 years or those already retired. The LEQ measures educational, occupational, and cognitive lifestyle activities at different stages of life. It is not suitable for people with a severe mental illness. In addition, it takes approximately 30 min, which makes it difficult to implement in clinical practice.

The CRQ [14] consists of eight items that measure different aspects of intellectual activity: schooling, training courses, parents’ schooling, the occupation performed throughout life, musical training, and language proficiency. In addition, it inquires about the approximate frequency of the cognitively stimulating activities. In spite of being a quick scale and widely used in dementia, it has the disadvantage of not valuing the different previous stages of life. The variety of intellectual, psychical, and leisure activities have not been taken into account, nor the type of courses carried out. Thus, it has never been tested in severe mental illness.

As far as the CRS [15] is concerned, it consists of 24 items that measure participation in cognitively stimulating activities throughout a person’s lifetime and is divided into three different life stages. The CRS is divided into four categories: activities of daily life, training, information, hobbies, and social life, and the mean score on each item is corrected for the possible effect of the additional period, called late adulthood, for elderly adults. The educational and professional attainments items are not included in the scale despite being frequently used in the measurement of CR [4,7,8,9,10,12,13,14,16].

Finally, the CRIq [16] includes 20 items divided into 3 sections: CRI—education, CRI—working activity, and CRI—leisure time. It does not value continued studies as an occupation (which is important for SMI patients), or the educational level reached. Instead, this scale values the number of years of education, which makes it not possible to differentiate between a person who has passed a particular level of studies and someone who has repeated a year of schooling and may not have passed. The working activity and leisure time sections record the years rounded off on a five-year scale (0–5–10–15–20), rounded up to the nearest 5-year period, and the years for which the frequency of leisure activities is to be stated are those of the person’s whole adult life, making it unsuitable for patients with a SMI. In addition, neither does it separately assess the different stages of life.

Overall, considering that the age of onset of the more prevalent SMI is usually during the final adolescence and young adulthood, the previous questionnaires are not adapted to the characteristics that should be assessed in a young population with an SMI. Critical issues when assessing CR on SMI populations might be benefited of considering, for example, whether the person is working or the maximum level reached at work, while also scoring training occupational programs. Other questionnaires do not value the accumulation of CR in different age stages, or items that have been considered relevant such as language trainings. Modifiable factors such as intellectual and leisure activities (and the variety and frequency of activities) have not always been considered.

The onset of a SMI supposes an interruption in life development at early stages that is closely related to the later functional outcomes. Furthermore, neuropsychiatric developmental disorders (such as the SMI) must be differentiated from other neurodegenerative pathologies when evaluating CR. Unlike severe brain damage or dementia, in cases of SMI, especially in schizophrenia, CR is considered to be mediated by the own nature of the pathology’s neurodevelopment [3], hence the necessity for a specific assessment instrument. Although assessing CR could benefit in the understanding of the two illnesses (neurodegenerative and neuropsychiatric), the fact that the onset of the SMI is many years before the onset of a neurodegenerative illness gives an add-on value on the field of SMI. People with SMI present a prolonged illness course and the application of personalized intervention on CR could, to a certain extent, alter the progression of the illness. Accordingly the Schizophrenia and the Bipolar and Depressive Disorders Units at the Hospital Clinic de Barcelona developed the Cognitive Reserve Assessment Scale in Health (CRASH), a specific instrument for the assessment of CR in SMI populations. This study aimed to develop and investigate the psychometric properties of the CRASH.

## 2. Materials and Methods

### 2.1. Sample

For the present study a sample of 100 patients with SMI and 66 healthy controls (HC) were recruited. Participants were enrolled through the Schizophrenia and the Bipolar and Depressive Disorders Units at the Hospital Clinic de Barcelona. Both Units belong to a University Hospital and collaborate in the context of the Spanish Biomedical Research Networking Center on Mental Health (CIBERSAM) [17]. HC were recruited via advertisement.

Inclusion criteria for all participants were (1) aged between 18 and 50 years old, (2) fluent Spanish, and (3) signed informed consent. In the case of patients, they should also present a diagnosis of severe mental illness (diagnosis of schizophrenia, schizoaffective disorders, and bipolar I or II, according to DSM-IV criteria). Exclusion criteria were (1) mental retardation according to DSM-IV criteria; (2) history of head trauma with loss of consciousness; (3) organic disease with mental repercussions; and (4) significant physical or neurological condition that could affect neuropsychological performance, substance abuse, or dependence in the last 12 months, and electroconvulsive therapy (ECT) within the preceding year. The patients were matched with HC by age (± 10%), gender, and parental socioeconomic status (SES) (± 1 level) determined using Hollingshead’s Two-Factor Index of Social Position [18]. The exclusion criteria for controls were the same as for the patients, with some additional criteria such as the presence of a current or past psychotic disorder, major depression or other serious psychiatric illnesses, or having a first-degree relative with a history of psychotic or affective disorders.

Patients were divided into two groups according to their diagnostic category. Diagnoses of schizophrenia and schizoaffective disorders were categorized into “non-affective”, whereas bipolar disorder I and II were grouped as “affective”.

This study was conducted in accordance with the ethical principles of the Declaration of Helsinki and Good Clinical Practice and the Hospital Clinic Ethics and Research Board. The research project was approved by the Clinical Research Ethics Committee of the Hospital Clínic of Barcelona (Protocol number ID: HCB/2016/0579, Date: April 2016). All participants provided written informed consent prior to their inclusion in the study.

### 2.2. Assessments

#### 2.2.1. Clinical and Sociodemographic Assessment

Clinical and sociodemographic data were systematically obtained for all participants. Diagnoses were determined with the Structured Clinical Interview for DSM (SCID-I and II) [19,20] according to DSM-IV criteria.

Patients were assessed with a total of three clinical scales: the Positive and Negative Syndrome Scale (PANSS) [21,22], the Young Mania Rating Scale (YMRS) [23,24], and the Montgomery–Asberg Depression Rating Scale (MADRS) [25,26]. On each scale, the items were summed to obtain a total score. Higher scores indicate greater severity of the assessed symptomatology.

#### 2.2.2. Functional Assessment

The functional outcome was assessed by means of the Functioning Assessment Short Test (FAST) [27] and the Global Assessment of Functioning (GAF) [28]. The FAST is a scale used to evaluate six functional domains (autonomy, occupational functioning, cognitive functioning, financial issues, interpersonal relationships, and leisure time). Higher scores indicate worse functioning. The GAF is a scale used to assess the severity of symptoms and the level of functioning, on a numeric scale from 1 to 100. Higher scores indicate a better functioning.

#### 2.2.3. Neuropsychological Assessment

The premorbid IQ was estimated using the Vocabulary subtest of the Wechsler Adult Intelligence Scale (WAIS-III) [29]. To assess verbal memory, the Verbal Learning Test Spain Complutense for adults (TAVEC) [30] was used. Sustained attention was tested with the Continuous Performance Test–II (CPT-II) [31], version 5, corrected by age and educational level. To assess processing speed, Trail Making Test-part A (TMT-A) [32] and the Digit Symbol-Coding Subtest of the WAIS-III were used. Working memory was assessed by the Letter–Number Sequencing Subtest of the WAIS-III. Verbal fluency was tested with the animal fluency test [33]. Higher scores correspond to better performance in all cognitive domains except for attention.

#### 2.2.4. Cognitive Reserve Assessment

To assess CR, the CRQ [14] and CRASH were used. In both scales, the higher the score, the greater the CR. The CRQ has been chosen over all the other listed instruments due to its short time of administration (2 min), which is more similar to CRASH (10 min) than other questionnaires; the fact that it is validated in the Spanish language; and the fact that it takes into account the educational level.

The CRASH was developed by the Schizophrenia and the Bipolar and Depressive Disorders Units at the Hospital Clinic de Barcelona in order to measure CR in SMI populations (see Appendix A). To generate potential items, an in-depth review of the scientific literature and existing measures [13,14,15,16] was initially carried out. Efforts were made to assess how to define more precisely the large blocks that make up the concept of CR (premorbid IQ, education/occupation, and leisure activities) and the constraints of each measure of CR available, taking into account that the reference population was people with a SMI. Once the first draft of CRASH was prepared, it was presented to twelve health professionals on mental health (eight psychologists and four psychiatrists) through discussion groups. They were asked to evaluate the items of the CRASH scale, including the suitability, clarity, and expression of the wording, and any items omitted that they considered relevant. The scale was modified and adapted to the different comments received (provided that at least 50% of the discussion group agreed). Finally, the discussion groups focused on the review of each of the measuring items. Items rated as essential, suitable, and clear by at least 80 percent of the health professionals were included; the other items were reformulated and re-rated, confirming the face validity.

The CRASH scale provides a global score and a score for each of the domains that form it (education, occupation, and intellectual and leisure activities). The scale’s maximum total score is 90, and it can be calculated using a formula, created with the intention that all domains have the same weighting in the final score. The score for each domain is obtained by adding the scores of the items it contains. For all scores, the higher the result, the better the level of CR.

Education: It measures the degree of schooling attained and passed by the subject and his parents. It also measures academic performance during childhood and adolescence and the number of languages in which the subject is able to maintain a conversation.

Occupation: It includes five different levels of working activities taking into account the occupation of the highest level (without work experience, unskilled and skilled work, professional occupation, and highly intellectual occupation) and five levels of training courses that must have been carried out in the last year to be scored.

Intellectual and leisure activities: The frequency and variety of four activities (physical, intellectual, artistic, and cultural) are counted over three life stages of the subject: childhood and adolescence, adulthood, and the previous year. To be considered an activity, it must be characteristic of the person’s life and it must have been performed within at least one year or academic year. Regarding frequency, a Likert-type scale of 0–3 points was used. In this section, sociability and withdrawal (type and quantity of relationships) are also evaluated.

A manual has been prepared including a semi-structured interview and a scoring guide that indicates how to score each item. Unlike a neurocognitive assessment, which should only be administered by a neuropsychologist, the CRASH can be carried out by professionals from different areas (nurses, psychologists, therapists, and medical doctors, among others), which facilitates its application. Furthermore, the time required to administer the test is approximately 10 min, which makes it highly feasible.

### 2.3. Statistical Analysis

Data were analyzed using Statistical Package for the Social Sciences (SPSS), version 18 (v for Windows, SPSS Inc., Chicago, IL, USA). Descriptive analyses were conducted using chi-square for categorical variables and Student’s test for continuous variables. Group differences were examined using unpaired t-tests for normally distributed variables, or using Mann–Whitney U tests for non-normal data. Significance level was set at *p* < 0.05.

The internal consistency reliability of the CRASH was assessed using Cronbach’s alpha. Concurrent validity for the CRASH and CRQ was assessed by examining Spearman’s correlation. Spearman’s correlation coefficient was also performed to examine the relationship between CRASH and CRQ, neuropsychological tests, psychosocial functioning, and clinical course.

Exploratory factor analysis (EFA) was applied to each items of the scale. The Keiser–Meyer–Olkin (KMO) test for sampling adequacy and Bartlett’s test for sphericity were performed to ensure that the EFA was adequate for principal component factor solution.

To test the predictive capacity of CRASH and CRQ for each group, a logistic regression analysis was conducted. Additionally, receiver operating characteristic (ROC) curves were analyzed to assess the ability of the CRASH and CRQ to distinguish subjects with a SMI from HC.

In order to help guide clinical interpretation, severity levels have been created based on diagnosis. The authors have divided the CRASH score into high and low in each group (healthy control, non-affective and affective patients). Subjects with a value above the median were considered to have a high CRASH score, and those with a score below median were assumed to have a low CRASH score [11,12,34], providing ease of interpretation that indicated where the score lay in comparison to the norm group.

## 3. Results

### 3.1. Sociodemographic Characteristics of the Sample

A total of 100 patients with a SMI and 66 HC were enrolled. A summary of the baseline sociodemographic and clinical characteristics of patients and HCs is shown in Table 1. Significant differences in functional outcomes (GAF and FAST) and neuropsychological outcomes (premorbid IQ, verbal memory, processing speed, and working memory) were found between patients and HC. There were no differences in terms of gender, age, SES, tobacco, cannabis use, and some cognitive performance variables such as sustained attention and verbal fluency.

When only analyzing the clinical group, there were significant differences in sociodemographic, clinical, functional, and neuropsychological variables among non-affective psychoses and affective disorders. The affective group was older, presented better clinical profile (positive, negative, general, and total PANSS) and performed better in different cognitive measures (premorbid IQ, verbal memory, and processing speed), except in sustained attention that performed worse (see Table 2).

### 3.2. Internal Consistency and Convergent Validity

Regarding inter-rater reliability of CRASH, there was agreement amongst 90% of health professionals, surpassing the two phases of reliability in all the tests. Thus, the inter-judge reliability study guarantees that the professionals carried out a correct application and correction of the tests.

The entirety of the CRASH score demonstrated adequate internal consistency (α = 0.903). The CRASH tool was significantly correlated with the CRQ in patients (*r_s_* = 0.838, *p* < 0.001) and in HC (*r_s_* = 0.816, *p* < 0.001) that persisted after controlling for potencial confounders such as premorbid IQ. When considering clinical groups amongst patients, this association was present for both non-affective psychotic disorder and affective disorders (*r_s_* = 0.827, *p* < 0.001, *r_s_* = 0.795, *p* < 0.001; respectively).

### 3.3. Factorial Analysis

The construct validity of CRASH was determined by conducting a factor analysis. The study of the internal structure of CRASH determined a four-factor structure, explaining 70.26% of total variance. The Kaiser–Meyer–Olkin measure of sampling adequacy (KMO) index was 0.728 (over 0.6), while Bartlett’s Test of Sphericity yielded an acceptable result (*p* < 0.001).

The principal components factor solution for CRASH is shown in Table 3. Factor I accounted for 36.23% of the variance and consisted of items related to “sociability”. Factor II accounted for 13.78% of the variance and consisted of “leisure activities”. Factor III accounted for 11.31% of the variance and consisted of “occupation”. Factor IV accounted for 8.95% of the variance and consisted of “education”.

### 3.4. CRASH Score Comparisons Among the Subject Samples and Severity Levels

Significant differences between the total CRASH score of patients and control groups have been found. The patient group obtained lower scores compared to the control group (39.82 ± 10.64 vs. 52.10 ± 9.40, *p* < 0.001). Between diagnostic groups, affective patients displayed higher CRASH scores than non-affective patients (44.78 ± 11.09 vs. 37.02 ± 9.25, *p* < 0.001). The affective patients showed lower CRASH scores than HC (*p* < 0.001). After performing a logistic regression to assess the predictive power of CRASH for each group (patients/controls), the model explained between 31.3% (Cox & Snell R Square) and 42.4% (Nagelkerke R Square) of the variance and correctly classified 78.9% of the cases (B = 0.131; *p* < 0.001; Exp(B) = 1.140). The predictive power of CRQ was also assessed and correctly classified 69.90% of the cases (B = 0.329; *p* < 0.001; Exp(B) = 1.389).

**Figure 1:**
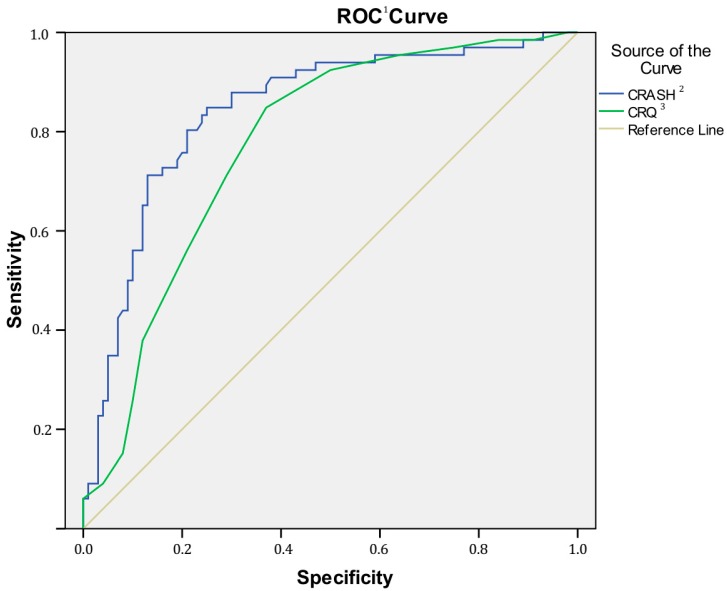
Curve between patients and controls (CRASH and cognitive reserve questionnaire CRQ). ^1^ ROC = receiver operating characteristic, ^2^ CRASH = Cognitive Reserve Assessment Scale in Health, ^3^ CRQ = Cognitive Reserve Questionnaire.

An ROC curve was generated to reveal the diagnostic accuracies of the CRASH and CRQ in discriminating patients with SMI from HC subjects. The analysis showed that CRASH, at a cutoff value of 47.58, had higher sensitivity (79%) and specificity (80.30%). The area under the curve was 0.842 (0.779–0.905) (see Figure 1). As far as the CRQ is concerned, at a cutoff value of 13.50, it had higher sensitivity (71.20%) and specificity (71%). The area under the curve was 0.773 (0.701–0.844).

### 3.5. Association Between CRASH and CRQ Scales and Clinical, Functionality, and Neuropsychological Test

Correlation analyses between CRASH and CRQ scales and clinical, functional, and neuropsychological outcomes are shown in Table 4. In the control group, the CRASH score was not associated with any measure. Nevertheless, the CRQ was related to some cognitive measures such as attention, processing speed, and premorbid IQ. In patients, the CRASH and CRQ total score were associated with clinical, functional, and almost all cognitive domains (except sustained attention). There were differences between non-affective psychotic disorders and affective disorders in these associations. In the non-affective psychosis group, the CRASH score was associated with premorbid IQ (*r_s_* = 0.387, *p* = 0.002), attention (*r_s_* = −0.302, *p* = 0.021), verbal fluency (*r_s_* = 0.504, *p* < 0.001), verbal memory (*r_s_* = 0.288, *p* = 0.026), processing speed (*r_s_* = 0.514, *p* = 0.014), GAF (*r_s_* = 0.292, *p* = 0.049), FAST (*r_s_* = −0.287, *p* = 0.048), and PANSS total score (*r_s_* = −0.318, *p* = 0.031). The CRQ was associated with age (*r_s_* = 0.294, *p* = 0.020) and three cognitive domains: verbal fluency (*r_s_* = 0.354, *p* = 0.006), verbal memory (*r_s_* = 0.217, *p* = 0.036), and premorbid IQ (*r_s_* = 0.500, *p* < 0.001). It was not associated with functionality or clinical outcomes in non-affective psychosis group. Instead, in patients with an affective disorder there were no associations between CRASH and neuropsychological, functional, and clinical outcomes, except for verbal memory (*r_s_* = 0.742, *p* < 0.001). The CRQ was also associated only with verbal memory (*r_s_* = 0.556, *p* < 0.001).

After dividing the CRASH into low or high level in each group (healthy control, non-affective, and affective patients), the differences on sociodemographic, clinical, functional, and neuropsychological variables were analyzed. In controls, there were no significant differences between those with higher and lower CRASH scores (above or below a score of 54). In the non-affective psychosis group, a significantly better performance was determined in patients with higher CRASH scores (above a score of 37) in all the cognitive domains evaluated, except for working memory, and lower negative and total symptoms assessed by PANSS scale. There were no differences in terms of age; gender; psychosocial functioning; and depressive, manic, or positive symptoms. In the affective disorder group, comparing those with high and low CRASH (above or below a score of 45), there were significant differences only in verbal memory performance (*p* < 0.001) (see Table 5).

An additional analysis was performed in order to clarify the necessity to divide the CRASH score in high and low above the median for each group, considering that subjects with a value above the cut-off point of 48 had a higher CRASH score. When comparing those with higher and lower CRASH scores, there were no significant differences in the non-affective psychosis group and in the affective disorder group there was only one significant difference for verbal memory performance (*p* < 0.001).

## 4. Discussion

This study confirmed the reliability and validity of the CRASH scale for patients with a SMI. The advantages of this new measure over existing measures include (a) the scale is adapted to the characteristics of this population, especially in the occupation domain, taking into account both training undertaken in the last year and the highest level occupation; (b) dividing it by periods of life facilitates the memory recall of the participants and provides information that, later, can be analyzed to know which period has been the most active in a person’s life and what kind of relationship it might have with other variables, such as cognitive impairment or psychosocial functioning; (c) it gives importance to modifiable factors such as intellectual and leisure activities; and (d) it is susceptible to being enhanced through psychosocial interventions.

Psychometric testing revealed excellent internal consistency and its statistically significant correlation with the CRQ confirms that the CRASH scale measures the intended construct of CR. The high association (0.8 and above) between CRASH and CRQ may be due to a high relationship between variables. However, as previously mentioned, it might be interesting to separately evaluate different activities and stages of life to explore the accumulation of CR and to know which period has been the most active in a person’s life, as well as which activities are most relevant. It could also be used to analyze what type of relationship CR might have with other variables, such as cognitive impairment. The principal components factor solution for CRASH extracted four factors: “education”, “occupation”, “leisure activities”, and “sociability”. These factors are foreseeable as the most common proposed CR indicator variables include educational level, occupational attainment, and leisure activities [4,8,9,10,12].

As expected, the CRASH score was higher for HC than for patients. This result is in accordance with previous studies that compared CR levels in patients and control subjects [4,8,10,12]. Amongst patients, the clinical group with an affective disorder showed a higher CRASH score compared to those with non-affective psychoses. This result is in line with our recently published study that explored the implications of CR in a FEP sample depending on whether the diagnosis was affective or non-affective FEP [12]. Indeed, the current findings suggest that the CRASH may have the ability to discriminate between patients with SMI and HC, with a high sensitivity (79%) and specificity (80.30%). The predictive power of CRASH is higher than CRQ (78.9% vs. 69.90%, respectively), indicating that the CRASH scale probably takes into account differential characteristics of the population with SMI.

The CR level was different between groups, and a serious limitation of norm-reference tests is that the reference group may not represent the population of interest. For this reason and in order to help guide clinical interpretation, the authors have divided the CRASH score into high and low in each group (HC, non-affective, and affective patients). The cut-off point value clearly differentiated patients from HC. However, it did not serve to differentiate non-affective patients with worse cognitive performance from those with higher cognitive performances.

In HC, the CRASH score was not associated with any measure and there were no differences between those with a higher CRASH score and those with a lower CRASH score. This is consistent with other studies that have shown that CR, after controlling for tobacco use, cannot predict cognitive performance nor functional outcomes [12]. As mentioned, one possible explanation of these results could be the CR concept itself [1,12]. HC are free from any pathology that might affect their cognitive performance or functionality, and CR refers to the brain’s capacity to face a pathology using alternative, or more efficient, cerebral networks in order to minimize symptoms [1]. Hence, we might not be able to detect the potential protective effects of CR in HC. Another possibility, taking into account that CRQ is associated with attention, processing speed and premorbid IQ, could be that the CRASH scale may be helpful in discriminating patients from healthy population. CRASH may have the ability to discriminate between patients with SMI and HC with higher sensitivity and specificity than CRQ. The predictive power of CRASH is also higher than CRQ, indicating that the CRASH scale probably takes into account differential characteristics of the population with SMI, especially for non-affective patients. This is advantageous for the evaluation of people with SMI, but it may not differentiate HC with greater cognitive abilities. Finally, it could be because, as observed by Stern, there are at least two types of reserve [2] (the use of brain networks or cognitive paradigms that are less susceptible to disruption or the use of brain structures or networks not normally used by individuals with intact brains in order to compensate for brain damage), and these may need to be measured differently.

In patient groups, as expected, associations between higher CRASH and CRQ scores and better clinical, functional, and cognitive performance could be confirmed. However, there were differences in the associations according to the diagnosis. CRQ was associated with better clinical, psychosocial, and cognitive functioning in patients; however, it was not associated with functionality or clinical outcomes in non-affective psychosis group. As far as the CRASH is concerned, it was associated with functional, clinical, and neuropsychological outcomes in non-affective psychotic disorders. The results suggested that people with a lower CRASH score had greater cognitive impairment and worse clinical and psychosocial functioning. Specifically, comparing those with higher and lower CRASH scores, a significantly better performance was determined in patients with higher CR on different cognitive measures (premorbid IQ, verbal memory, sustained attention, processing speed, and verbal fluency) and lower symptom severity, especially the negative ones. These results are in line with previous studies [4,10,12,35,36,37] indicating that non-affective patients with lower CRASH scores could benefit from a cognitive rehabilitation program [38], oriented to improve performance in cognitive domains. This recommendation was already mentioned in our study of first psychotic episodes [12], specifically in those with a non-affective psychosis. In affective disorders, having a lower or higher level of CRASH was only associated with verbal memory performance. Some studies have shown that CR was significantly predictive of functioning and different cognitive domains [8,9,11], while others have reported that capacity only for verbal memory [12]. In fact, we have found a strong correlation between verbal memory and CRASH in affective patients, probably indicating the important role of this cognitive domain in the CR and as a ‘core’ deficit of bipolar disorder, even in the early stages of the illness [39,40,41,42]. There are aspects to keep in mind that could explain these differences such as the variables used to estimate the CR or the sample size. As recommended in previous reports from our group [12], for patients with affective disorders and lower CRASH scores a functional remediation therapy [43] can be suggested, since it is the intervention that has been shown to be the most effective in improving psychosocial functioning and verbal memory, the only altered domain found in our study [44,45]. Regardless of diagnosis, we consider that although there are important premorbid variables in the CR concept that are difficult to modify (such as premorbid IQ or education), interventions centered on CR stimulation and engaging lifestyle (such as physical, social, or leisure activities) could be beneficial to preventing or reducing the impact of illness [12,46,47], especially those conducted in the early stages of the illness, or even in people with a high risk of suffering psychosis.

Thus, with these preliminary results, authors believe that the CRASH scale may aid in the classification of patients while helping in the implementation of personalized interventions focused on improving core symptoms of SMI [46,47,48]. Is expected that it will allow for the homogeneous assessment of the concept of CR in the psychiatric population, and that this will be translated into more consistent, uniform, and replicable data. Further research should be conducted to use the CRASH scale as a stratification tool and a potential sensitive measure of change. There are already neuropsychological and functional interventions aimed at improving cognitive and psychosocial functioning, but until now the impact of intervening on the different areas of CR such as social, mental, physical activities, and hobbies in people with SMI has not been explored.

Some limitations of our work have to be taken into consideration before translating these findings into clinical practice. Firstly, the difference between the clinical sample size of non-affective and affective psychotic disorder groups (62 vs. 38). The small sample size may have interfered with the results (low statistical power), and further larger studies are required to confirm these findings. Secondly, executive function has not been assessed with specific tests, and we have used verbal fluency as a variable that expresses executive processes. The final limitation would be the cross-sectional study designs, which does not allow one to measure stability. However, it is not expected to change without an intervention. Despite these limitations, our study has significant strengths. We have developed a new instrument that measures CR level in people with SMI and can improve our understanding of individual differences in the course of SMI helping the implementation of personalized interventions.

## 5. Conclusions

In summary, CRASH showed strong internal consistency. It is the first measure developed specifically for patients with severe mental illness with optimal psychometric properties, facilitating reliable and valid measurement of cognitive reserve. The scale may aid in the stratification of patients and the implementation of personalized interventions.

## Figures and Tables

**Table 1:** Sociodemographic, functional, clinical, and neuropsychological variables for patients with severe mental illness and healthy controls.

	Patients (*n* = 100)	Healthy Controls (*n* = 66)	Statistic	*p*-Value
Gender: Male N (%)	65 (65)	35 (55)	*χ*^2^ = 2.378	0.084
Age ($\bar{X}$ ± SD)	32.68 ± 8.59	30.12 ± 8.15	*t* = 1.720	0.087
SES ^1^ (%)			*χ*^2^ = 5.967	0.309
High	30 (30)			
Medium-High	23 (23)			
Medium	19 (19)			
Medium-Low	19 (19)			
Low	7 (7)			
Missing value	2 (2)			
Tobacco use: Yes N (%)	39 (39)	19 (29)	*χ*^2^ = 2.542	0.076
Cannabis use: Yes N (%)	11 (11)	10 (15)	*χ*^2^ =0.372	0.352
**Functional variables (** $\bar{X}$ **± SD)**				
GAF ^2^ score	74.77 ± 11.94	90.79 ± 6.01	U = 228.50	**<0.001**
FAST ^3^	18.64 ± 13.22	2.66 ± 6.24	U = 392.00	**<0.001**
**Clinical variables (** $\bar{X}$ **± SD)**				
PANSS ^4^ positive	10.54 ± 4.25	NA ^8^		
PANSS negative	15.90± 6.20	NA		
PANSS general	26.82 ± 8.29	NA		
PANSS total	53.25 ± 16.89	NA		
YMRS ^5^ score	0.84 ± 1.81	NA		
MADRS ^6^ score	3.90 ± 4.82	NA		
**Neuropsychological performance (** $\bar{X}$ **± SD)**				
Premorbid IQ ^7^	101.87 ± 11.85	108.56 ± 9.48	*t* = −3.771	**<0.001**
Verbal memory	41.88 ± 11.45	55.25 ± 8.48	U = 1217.50	**<0.001**
Sustained attention	47.22 ± 13.26	45.91 ± 10.87	*t* = 0.621	0.535
Processing speed	48.51 ± 11.78	61.73 ± 6.27	U = 134.50	**<0.001**
Working memory	47.15 ± 11.96	51.98 ± 9.03	U = 2188.00	**0.011**
Verbal fluency	47.60 ± 15.83	44.29 ± 17.28	*t* = −1.268	0.209

^1^ SES = Socioeconomic status, ^2^ GAF = Global Assessment of Functioning, ^3^ FAST = Functioning Assessment Short Test, ^4^ PANSS = Positive and Negative Symptom Scale, ^5^ YMRS = Young Mania Rating Scale, ^6^ MADRS = Montgomery–Asberg Depression Rating Scale, ^7^ IQ = Intelligence Quotient, ^8^ NA = Not applicable. Significant differences (*p* < 0.05) marked in bold.

**Table 2:** Baseline sociodemographic, functional, clinical, and neuropsychological performance for patients (affective vs. non-affective).

	Patients (n = 100)		Statistic	*p*-Value
	Non-Affective (*n* = 62)	Affective (*n* = 38)		
Gender: Male N (%)	40 (64.52)	25 (65.79)	*χ*^2^ = 0.009	0.551
Age ($\bar{X}$ ± SD)	30.82 ± 7.74	35.71 ± 9.13	*t* = −2.578	**0.011**
SES ^1^ N (%)			*χ*^2^ = 7.706	0.173
High	21 (34)	9 (24)		
Medium-High	13 (21)	10 (26)		
Medium	11 (18)	8 (21)		
Medium-Low	11 (18)	8 (21)		
Low	6 (9)	1 (3)		
Missing value	0 (0)	2 (5)		
Age of onset	25.67 ± 6	25.00 ± 7	*t* = 0.489	0.626
Tobacco use: Yes N (%)	22 (35)	16 (42)	*χ*^2^ = 0.201	0.407
Cannabis use: Yes N (%)	7 (11)	4 (11)	*χ*^2^ = 0.077	0.526
**Functional variables (** $\bar{X}$ **± SD)**				
GAF ^2^ score	74.04 ± 11.66	78.57 ± 7.45	*t* = −1.366	0.177
FAST ^3^	19.19 ± 14.34	16.61 ± 11.41	*t* =0.838	0.405
**Clinical variables (** $\bar{X}$ **± SD)**				
PANSS ^4^ positive	11.43 ± 4.48	7.86 ± 1.61	U = 127.00	**0.001**
PANSS negative	17.24 ± 6.07	11.07 ± 3.87	U = 132.00	**0.001**
PANSS general	28.11 ± 8.58	22.71 ± 6.21	*t* = 2.180	**0.033**
PANSS total	56.76 ± 17.13	41.64 ± 10.60	U = 147.50	**0.002**
YMRS ^5^ score	0.73 ± 2.20	1.26 ± 1.56	*t* = −1.150	0.253
MADRS ^6^ score	5.17 ± 6.15	3.68 ± 3.00	U = 779.00	0.899
**Neuropsychological performance (** $\bar{X}$ **± SD)**				
Premorbid IQ ^7^	98.73 ±11.63	107.16 ± 10.35	*t* = -3.515	**0.001**
Verbal memory	38.42 ± 10.16	47.06 ± 12.40	U = 696.50	**0.002**
Sustained attention	44.77 ± 12.27	51.10 ± 14.07	*t* = −2.151	**0.034**
Processing speed	39.85 ± 9.54	54.94 ± 8.84	*t* = −5.996	**<0.001**
Working memory	45.55 ± 12.32	49.78 ± 11.01	*t* = −1.713	0.090
Verbal fluency	45.55 ± 16.88	46.71 ± 14.06	*t* = −0.227	0.821

^1^ SES=Socioeconomic status, ^2^ GAF= Global Assessment of Functioning, ^3^ FAST=Functioning Assessment Short Test, ^4^ PANSS= Positive and Negative Symptom Scale, ^5^ YMRS= Young Mania Rating Scale, ^6^ MADRS= Montgomery-Asberg Depression Rating Scale, ^7^ IQ= Intelligence Quotient. Significant differences (*p* < 0.05) marked in bold.

**Table 3:** Principal Components Factor Solution for Cognitive Reserve Assessment Scale in Health (CRASH).

Factor I		Factor II		Factor III		Factor IV	
Sociability	LDG ^1^	Leisure Activities	LDG	Occupation	LDG	Education	LDG
10. Social activity in childhood and adolescence	0.867	7. Leisure activities in childhood and adolescence	0.802	5. Training	0.762	1. Education	0.726
11. Social activity in adulthood	0.908	8. Leisure activities in adulthood	0.917	6. Employment	0.890	2. Educational attainment of parents	0.697
12. Social activity in the past year	0.923	9. Leisure activities in the past year	0.874			3. Scholastic performance	0.512
						4. Languages	0.558

^1^ LDG = Loading.

**Table 4:** Spearman’s correlation analyses of CRASH and CRQ with functional, clinical, and cognitive outcomes.

	Patients				Healthy Controls			
	Spearman’s Correlation CRASH ^1^	*p*-Value	Spearman’s Correlation CRQ ^2^	*p*-Value	Spearman’s Correlation CRASH	*p*-Value	Spearman’s Correlation CRQ	*p*-Value
**Functional variables (** $\bar{X}$ **± SD)**								
GAF ^3^	0.275	**0.033**	0.309	**0.016**	−0.077	0.628	−0.100	0.53
FAST ^4^	−0.298	**0.007**	−0.376	**0.001**	−0.026	0.838	−0.086	0.51
**Clinical variables (** $\bar{X}$ **± SD)**								
PANSS ^5^ positive	−0.333	**0.002**	−0.455	**<0.001**	NA			
PANSS negative	−0.277	**0.032**	−0.333	**0.009**				
PANSS general	−0.212	0.10	−0.274	**0.034**				
PANSS total	−0.280	**0.030**	−0.363	**0.004**				
YMRS ^6^ score	− 0.070	0.376	−0.058	0.61				
MADRS ^7^ score	−0.047	0.369	−0.150	0.18				
**Neuropsychological performance (** $\bar{X}$ **± SD)**								
Premorbid IQ ^8^	0.405	**<0.001**	0.427	**<0.001**	0.208	0.105	0.267	**0.036**
Verbal memory	0.533	**<0.001**	0.443	**<0.001**	0.222	0.088	0.077	0.56
Sustained attention	−0.117	0.27	−0.021	0.84	−0.089	0.515	−0.340	**0.010**
Processing speed	0.397	**0.003**	0.359	**0.010**	0.425	0.062	0.477	**0.033**
Working memory	0.205	**0.045**	0.199	0.05	0.227	0.081	0.250	0.054
Verbal Fluency	0.325	**0.002**	0.250	**0.016**	−0.347	0.114	0.023	0.87

^1^ CRASH=Cognitive Reserve Assessment Scale in Health, ^2^ CRQ= Cognitive Reserve Questionnaire, ^3^ GAF= Global Assessment of Functioning, ^4^ FAST=Functioning Assessment Short Test, ^5^ PANSS= Positive and Negative Symptom Scale, ^6^ YMRS= Young Mania Rating Scale, ^7^ MADRS= Montgomery-Asberg Depression Rating Scale, ^8^ IQ= Intelligence Quotient. Significant differences (*p* < 0.05) marked in bold.

**Table 5:** Sociodemographic, clinical, functional, and cognitive performance among subjects with high and low CRASH scores.

	Non-Affective (*n* = 62)			Affective (*n* = 38)		
	Low CRASH (<37)	High CRASH (≥37)	*p*	Low CRASH (<45)	High CRASH (≥45)	*p*
**Sociodemographic variables**						
Gender: Male *N* (%)	20 (65)	20 (65)	0.64	14 (67)	11 (65)	0.74
Age ($\bar{X}$ ± SD)	29.77 ± 8.07	31.87 ± 7.38	0.29	35.76 ± 9.61	35.65 ± 8.79	0.97
SES ^1^ N (%)			0.43			0.19
High	9 (28)	12 (40)		4 (27)	5 (24)	
Medium-High	5 (17)	8 (26)		4 (27)	6 (29)	
Medium	6 (17)	6 (19)		3 (20)	5 (24)	
Medium-Low	8 (24)	3 (11)		3 (20)	6 (24)	
Low	5 (15)	1 (4)		1 (7)	0 (0)	
Missing value	0 (0)	0 (0)		1 (7)	1 (5)	
Age of onset	25.17 ± 5.88	26.11 ± 5.31	0.55	23.95 ± 6.07	25.93 ± 8.85	0.43
**Functional variables** ($\bar{X}$ ± SD)						
GAF ^2^	70.55 ± 11.04	76.73 ± 11.61	0.06	79.50 ± 7	76.25 ± 8	0.48
FAST ^3^	23.41 ± 14.34	15.62 ± 13.60	0.07	18.55 ± 12	13.50 ± 10	0.23
**Clinical variables** ($\bar{X}$ ± SD)						
PANSS ^4^ positive	12.71 ± 5.47	10.36 ± 3.16	0.07	8.11 ± 1.97	7.40 ± 0.55	0.45
PANSS negative	19.38 ± 6.07	15.44 ± 5.58	**0.027**	11.56 ± 4.25	10.26 ± 3.35	0.55
PANSS general	30.33 ± 9.44	26.24 ± 7.46	0.10	23.22 ± 6.44	21.80 ± 6.38	0.69
PANSS total	62.43 ± 18.63	52 ± 14.47	**0.038**	42.89 ± 11.25	39.40 ± 10.11	0.58
YMRS ^5^	1.04 ± 3.00	0.46 ± 1.17	0.37	1.52 ± 1.67	0.85 ± 1.35	0.23
MADRS ^6^	5.32 ± 5.78	5.04 ± 6.56	0.88	3.76 ± 2.95	3.54 ± 3.21	0.83
**Neuropsychological performance** ($\bar{X}$ ± SD)						
Premorbid IQ ^7^	93.61 ± 11	103.84 ± 10	**<0.001**	105.71 ± 11.10	108.94 ± 9.37	0.35
Verbal memory	34.07 ± 8.43	42.50 ± 10.06	**<0.001**	37.90 ± 11.18	53.26 ± 7.82	**<0.001**
Sustained attention	48.68 ± 11.75	41.12 ± 11.78	**0.018**	51.80 ± 14.86	50.26 ± 13.50	0.75
Processing speed	34.50 ± 9.35	43.29 ± 8.23	**0.027**	54.03 ± 7.48	55.67 ± 9.99	0.61
Working memory	43.24 ± 11.82	47.86 ± 12.57	0.14	48.56 ± 11.45	51.31 ± 10.59	0.46
Verbal fluency	39.76 ± 16.91	51.35 ± 14.99	**0.008**	45.55 ± 16.77	48.37 ± 9.25	0.57

^1^ SES=Socioeconomic status, ^2^ GAF= Global Assessment of Functioning, ^3^ FAST=Functioning Assessment Short Test, ^4^ PANSS= Positive and Negative Symptom Scale, ^5^ YMRS= Young Mania Rating Scale, ^6^ MADRS= Montgomery-Asberg Depression Rating Scale, ^7^ IQ= Intelligence Quotient. Significant differences (*p* < 0.05) marked in bold.
